# Cofactor Independent Phosphoglycerate Mutase of *Brugia malayi* Induces a Mixed Th1/Th2 Type Immune Response and Inhibits Larval Development in the Host

**DOI:** 10.1155/2014/590281

**Published:** 2014-07-01

**Authors:** Prashant K. Singh, Susheela Kushwaha, Ajay K. Rana, Shailja Misra-Bhattacharya

**Affiliations:** Division of Parasitology, CSIR-Central Drug Research Institute, B.S. 10/1, Sector 10, P.O. Box 173, Jankipuram Extension, Sitapur Road, Lucknow, Uttar Pradesh 226031, India

## Abstract

Lymphatic filariasis is a major debilitating disease, endemic in 72 countries putting more than 1.39 billion people at risk and 120 million are already infected. Despite the significant progress in chemotherapeutic advancements, there is still need for other measures like development of an effective vaccine or discovery of novel drug targets. In this study, structural and immunological characterization of independent phosphoglycerate mutase of filarial parasite *Brugia malayi* was carried out. Protein was found to be expressed in all major parasite life stages and as an excretory secretory product of adult parasites. Bm-iPGM also reacted to all the categories of human bancroftian patient's sera including endemic normals. *In vivo* immunological behaviour of protein was determined in immunized BALB/c mice followed by prophylactic analysis in BALB/c mice and *Mastomys coucha*. Immunization with Bm-iPGM led to generation of a mixed Th1/Th2 type immune response offering 58.2% protection against larval challenge in BALB/c and 65–68% protection in *M. coucha*. *In vitro* studies confirmed participation of anti-Bm-iPGM antibodies in killing of *B. malayi* infective larvae and microfilariae through ADCC mechanism. The present findings reveal potential immunoprotective nature of Bm-iPGM advocating its worth as an antifilarial vaccine candidate.

## 1. Introduction

Lymphatic filariasis (LF) is one of the oldest and most morbid and debilitating parasitic diseases [1] caused by three thread-like nematode worms,* Wuchereria bancrofti*,* Brugia malayi*, and* B. timori*. An estimated 130 million people in 72 countries are currently infected, and around 1.34 billion are at risk of infection [2, 3]. Diethylcarbamazine (DEC), principally microfilaricidal, is the only drug of choice for the treatment of LF [4]. Mass drug administration (MDA) using DEC, ivermectin (IVN), and albendazole (ALB) combination has been quite successful; however, several rounds of MDA are required to reduce the level of infection to sustain transmission [5]. As no drug is available against adult parasite, alternative strategies like vaccine development need to be explored in order to improve the effectiveness of disease control and prevention programmes. With the availability of the draft genome sequence of* B. malayi *[6] and the sequencing of endosymbiont* Wolbachia*, mosquito vectors (*Aedes* and* Anopheles*), and the human host, the ability to carry out large-scale comparative genomics presents opportunities to understand the molecular basis of parasitism defining molecules and pathways unique to nematode development and parasitism that can be characterised as the novel antifilarial drug targets or vaccine candidates.

In the current study, molecular, biophysical, and immune characterisation of independent phosphoglycerate mutase (Bm-iPGM) of human filarial parasite* B. malayi* was carried out. Phosphoglycerate mutases, the key enzymes in the glycolytic and gluconeogenic pathways, exist in two different forms having different mechanism of action and structure and that are either cofactor (2,3-diphosphoglycerate) dependent or cofactor-independent. The independent form is predominant in plants, nematodes, bacteria, and archaea [7]. All experimentally characterised iPGMs from eubacteria, plants, and invertebrates are monomers with a molecular mass of 55–75 kDa [8, 9]. The absence of iPGM from humans and being indispensable in all nematodes including the filariids [10] advocate its potential as anthelminthic drug target. Bm-iPGM was purified successfully using bacterial host* E. coli*. Circular dichroism (CD) and fluorescence spectra of the recombinant protein were obtained to determine its secondary structure and native conformation. The abundant sharing of Bm-iPGM by all the major life-forms of* B. malayi* and its release in the form of excretory-secretory products [11] pointed towards its immunogenic nature.* In silico *analysis of Bm-iPGM predicted it to be highly antigenic with the presence of both MHC I and MHC II binding peptides. The antigenic nature was further validated by the cross-reactivity of Bm-iPGM with human bancroftian sera of different categories of LF which persuaded us to examine the immunoprophylactic efficacy of the recombinant protein in animal models of LF.

*In vivo* immune characterisation of Bm-iPGM in BALB/c mice revealed it to invoke a mixed type of Th1/Th2 immune response. The immunised animals (BALB/c and* Mastomys*) that were challenged with the infective larvae displayed reduced worm establishment. As active filarial infections are accompanied with downregulation of the host immune system, skewing the helper immune response of host to Th2 type, we propose that immunization with Bm-iPGM is capable of generating a mixed Th1/Th2 type response that is unfavourable for parasite establishment and was responsible for providing considerable protection against LF in mouse models thus validating Bm-iPGM to be possible vaccine candidate against LF.

## 2. Materials and Methods

### 2.1. Experimental Animals

Randomly bred, 6–8-week-old male BALB/c (35) and* Mastomys coucha* (36) were used in the experiment. The animals were maintained in proper housing condition at Laboratory Animal Facility at CSIR-Central Drug Research Institute (CDRI), Lucknow, India. Animals were fed on standard pellet diet and water* ad libitum*. The animals and the animal experimental procedures were approved by the Animal Ethics Committee of CDRI duly constituted under the provisions of CPCSEA (Committee for the Purpose of Control and Supervision on Experiments on Animals), Government of India. The study bears the IAEC number 83/09/Para/IAEC dated 27/04/09. All the experiments were performed in duplicate (both for* Mastomys* and BALB/c) and almost similar results were obtained in both the experiments and, therefore, pooled.

### 2.2. Parasites

Infective larvae (L3) of* B. malayi* were recovered from the laboratory bred vector mosquitoes (*Aedes aegypti*) fed on donor* Mastomys* 9 ± 1 day back [12]. L3 were isolated from gently crushed mosquitoes by Baermann technique, washed, and counted in Ringer's solution. Adult* B. malayi* worms and microfilariae (Mf) were collected from the peritoneal cavities of the infected jirds on day 80–85 after L3 inoculation.

### 2.3. Homology Modelling of Bm-iPGM and Amino Acid Sequences

The homology model for Bm-iPGM was generated using Phyre server [13]. Bm-iPGM structure was generated with 100% precision and 41% identity using structure of* Bacillus anthracis* cofactor-independent 2 phosphoglycerate mutase as template (PDB id: c2ifyA, length: 508 AA). The data generated was analysed by The PyMOL Molecular Graphics System, Version 1.3, Schrödinger, LLC, and the cartoon structure was generated. Amino acid sequence of Bm-iPGM was also aligned with iPGM from* B. anthracis *(accession number: 2IFY_A) using Clustal W programme.

### 2.4. *In Silico* Antigenicity Prediction

The antigenicity of Bm-iPGM was determined by Kolaskar and Tongaonkar method [14]. This semiempirical method predicts antigenic determinants based on the physicochemical properties of amino acid residues and the frequencies of their occurrence in experimentally known segmental epitopes. Prediction of immunodominant T cell antigenic sites from the primary sequence of Bm-iPGM was determined by ProPred-I and ProPred MHC class-II binding peptide prediction servers, which are online web tools for the prediction of peptide binding to MHC class-I (HLA-A1, HLA-A2, HLA-A0201, HLA-A0205, HLA-A1101, HLA-A3101, HLA-A3302, HLA-B2102, HLA-A3501, HLA-A4403, and HLA-5101) and class II (HLA-DRB1_0101, HLA-DRB1_0301, HLA-DRB1_0401, HLA-DRB1_0701, and HLA-DRB1_0801) alleles [15, 16]. The highest ranking MHC I and MHC II binding peptides were highlighted in the cartoon structure of Bm-iPGM obtained earlier.

### 2.5. Cloning, Expression, and Purification of Bm-iPGM

Expression and purification of Bm-iPGM was done as described elsewhere with minor modifications [17]. Briefly, gene specific forward (5′AGTCGGATCCATGGCCGAAGCAAAGAATCG-3′) and reverse (5′ATGCCTCGAGGGCTTCATTACCAATGGC3′) primers having restriction sites for the enzymes* BamHI *(F) and* XhoI* (R) were synthesised. Amplification of gene was carried out using 1 *μ*M of each primer, 200 *μ*M of each dNTP (Fermentas, USA), 0.5 unit taq DNA polymerase (Invitrogen, USA), 1xPCR buffer, and 1.5 *μ*M of MgCl_2_ with the following reaction conditions: 95°C for 2 min, followed by 29 cycles of 95°C for 1 min, 58°C for 1 min, 72°C for 2 min, and 1 cycle at 72°C for 10 min. The 1548 bp amplified gene was cloned into pTZ57R/T (2886 bp) vector as per manufacturer's instructions (Fermentas, USA). Plasmid DNA was isolated, and the insert was verified by sequencing. The 1548 bp gene was subcloned into pET28a expression vector for protein expression and purification. The conditions like temperature, isopropyl *β*-D-thiogalactopyranoside (IPTG) concentration, and duration of induction were standardized for optimal expression of the protein in soluble form. Five mL cultures (*E. coli* strain BL21 (DE3)) were grown at 37°C in an incubator shaker at 220 rpm and induced (at OD_600_ of 0.5-0.6) for 4 h with 0.2, 0.5, and 1.0 mM IPTG. After induction, cells were harvested by centrifugation at 7000 rpm for 5 min and lysed in 5 mL sample buffer (0.313 M Tris-HCl, pH 6.8, 50% glycerol, 10% SDS, and 0.05% bromophenol blue) for analysis on 10% SDS-PAGE (Laemmli 1970) along with uninduced vector control culture. To observe the solubility of recombinant protein, the cell pellet was resuspended in 1 mL of lysis buffer (50 mM Tris-HCl, pH 7.5; 200 mM NaCl; and 100 mM DTT), sonicated at 10 db/10s in a Soniprep 150 sonicator in cold. The cell lysate was centrifuged at 14,000 rpm for 30 minutes to collect the supernatant containing soluble fraction and the remaining pellet containing insoluble fraction was resuspended in 1 mL of lysis buffer. Soluble and insoluble fractions were then analyzed in parallel on 10% SDS-PAGE and visualized by Coomassie blue staining. For purification of the expressed recombinant protein, the supernatant was run on Ni-NTA agarose affinity column preequilibrated with 50 mM Tris-HCl buffer (pH 7.5) along with 200 mM NaCl and 10 mM imidazole. The column was subsequently washed with the same buffer containing 25 mM and 40 mM imidazole and the recombinant protein was eluted with 250 mM imidazole, analyzed on SDS-PAGE and protein expression was confirmed using anti-His antibody in Western blot. Following purification, the exact mass of Bm-iPGM was determined through matrix-assisted laser desorption/ionization time-of-flight mass spectrometry (MALDI-TOF). The protein was dialysed in water (O/N) to remove any buffer content and diluted in 30% acetonitrile (ACN) and 0.1% trifluoroacetic acid (TFA) solution in a matrix of sinapinic acid. Ten *μ*L of the prepared sample was then pipetted on the plate and was air-dried to allow cocrystallization of the protein and the matrix; the target plate was loaded in the MALDI-TOF spectrometer (Applied Biosystems MDS Sciex 4800 Plus MALDI TOF/TOF, Foster City, CA, USA). To check whether the recombinant protein was biochemically active, the activity of recombinant Bm-iPGM was measured (data not shown) as described previously using a standard one-step enzyme-coupled assay [17].

### 2.6. Biophysical Investigations

CD measurements were made on JASCO J810 Spectropolarimeter calibrated with ammonium (+)-10-camphorsulfonate with 6 *μ*M protein in 10 mM citrate, glycine, and HEPES (CGH) buffer of desired pH with a 2 mm path length cell at 25°C using the following parameters: 1 sec response, 50 nm/min scan speed, 0.1 nm data acquisition interval, 3 accumulations, and 2 nm bandwidth. The spectra were recorded over a range of 190–250 nm. The values obtained were normalized by subtracting the baseline recorded for the buffer under similar conditions. The ellipticity was reported as molar ellipticity (*θ*) (mdeg·cm^−2^·mol^−1^). Fluorescence spectra were recorded with Perkin Elmer LS50B luminescence spectrometer in a quartz cell of 5 mm path length. 6 *μ*M concentration of protein in 50 mM phosphate buffer (pH 7.0) was incubated at 25°C, before recording the spectra. Excitation wavelength was 280 nm and the spectra were recorded between 290 nm and 400 nm.

### 2.7. Production of Polyclonal Antibodies to Analyze Stage Specific Expression of Bm-iPGM

Five BALB/c mice were administered subcutaneously with the recombinant Bm-iPGM (25 *μ*g/animal) in three doses at 2-week intervals. First dose was given in Freund's complete adjuvant (FCA), while the remaining two in Freund's incomplete adjuvant (FIA). The animals were euthanized a week after the last protein booster and blood was collected for serum separation. For preparation of soluble extracts, adult parasites, L3, and Mf were homogenized in sterile PBS (pH 7.2) containing protease inhibiter cocktail (Sigma, USA) in cold and left for overnight (O/N) extraction at 4°C and were further sonicated and centrifuged. The protein content was estimated in the supernatant by Bradford method [18]. Protein was loaded on to 10% SDS-PAGE and transferred to nitrocellulose membrane (NC). Membrane strips were incubated with Bm-iPGM specific polyclonal antibody raised in mouse (1 : 5000). After washing, membranes were incubated with HRP-goat anti-mouse antibody and were developed with substrate o-phenylenediamine dihydrochloride (OPD) [19, 20]. Bm-iPGM gene expression in various stages of* B. malayi* was also observed using cDNA. Adult worms, L3, and Mf were recovered as mentioned above. RNA was extracted from all the three life stages using TRIzol reagent (Invitrogen, USA) and quantified with a GeneQuant (Bio-Rad). After treatment with DNase I to eliminate genomic DNA contamination, 2 *μ*g of total RNAs from each life stage was used for the first cDNA synthesis using a first-strand cDNA synthesis kit (Invitrogen, USA). cDNAs were amplified with specific primer pairs under the conditions mentioned above.

### 2.8. Analysis of Bm-iPGM in Excretory and Secretory (ES) Product

Adult worms (4 worms/mL) were maintained* in vitro* in serum-free RPMI 1640 (GIBCO) supplemented with antibiotic antimycotic (Invitrogen, 100 U/mL penicillin, 100 mg/mL streptomycin, and 0.25 mg/mL of amphotericin B) and 25 mM HEPES at 37°C in 5% CO_2_ in air. The utilized media was collected and replaced with fresh medium every 24 h continuously up to 7 days. The medium collected was filtered through 0.2 mM filters (Millipore, USA) and stored, pooled, and concentrated using 3 kDa cut-off membranes filters (Millipore, USA). Concentrated ES product and recombinant Bm-iPGM were individually run on 10% SDS-PAGE and transferred to NC membrane. Membrane was blocked with 3% skimmed milk for 1 h and incubated at room temperature (RT) with 1 : 200 dilution of anti-Bm-iPGM antibodies raised in BALB/c mice. The membrane was reincubated with goat 1 : 10,000 dilution of anti-mouse IgG-HRP antibody for 1 h at RT and the reaction was developed with the substrate 3,3-diaminobenzidine (DAB) tetrahydrochloride.

### 2.9. Reactivity of Bm-iPGM with Human Bancroftian Antibodies

Reactivity of recombinant enzyme was observed with the antibody present in the sera of human subjects by Western blotting and ELISA. For serum, blood was collected from* W. bancrofti* endemic area in the outskirts of Lucknow, India, and was categorized as endemic normal, asymptomatic microfilaria carriers, microfilaraemic symptomatic, and amicrofilaraemic symptomatic. Sera from humans living in filaria free zones like Jammu and Kashmir, India, served as nonendemic control. Mf presence or absence was earlier determined in the 2 mL night blood by membrane filtration technique [21]. Purified recombinant protein along with prestained molecular weight marker was run on a preparative 10% SDS-PAGE, transferred to NC membrane, and processed for immune-recognition with human sera pools (1 : 200) of 10 subjects per category. Goat anti-human IgG-HRP (1 : 10,000 dilutions) was used as secondary antibody and reaction was developed by the DAB substrate.

IgG antibodies in individual sera sample (10 sera of each category) belonging to microfilaraemic, amicrofilaraemic symptomatic, endemic normal (EN), and nonendemic normal (NEN) categories were measured using recombinant Bm-iPGM as an antigen in ELISA as stated above. The human sera samples were added at 1 : 200 dilutions as primary antibody while goat anti-human IgG antibody-HRP (1 : 10,000) was used as secondary antibody.

### 2.10. Immunization of BALB/c and* Mastomys* with Recombinant Bm-iPGM

Immune characterization of recombinant Bm-iPGM was carried out in BALB/c while prophylactic efficacy was investigated both in BALB/c and in* Mastomys*. BALB/c mice do not support full development of L3 to preadult or adult stage nor develop microfilaraemia while* Mastomys* being highly susceptible supports full development from L3 to adult with the release of Mf. Mice are ideal for immune characterization of an antigen. We divided the animals into three different treatment groups (ten BALB/c mice and twelve* Mastomys* per group were used) which received three equal immunization doses on day 0, day 15, and day 23. Animals in treatment group 1 received only PBS (unimmunized control group), while animals in treatment group 2 received equivalent volume of FCA (day 15) and FIA (day 23) in PBS (adjuvant group). Animals in the last treatment group 3 were administered with 25 *μ*g recombinant protein along with the adjuvant (FCA on day 15 and with FIA on day 23), respectively. Preimmunized sera were collected from the retroorbital plexus of each mouse prior to immunization and thereafter on days 14 and 20 after first antigen dose. One week following final booster dose, half of the BALB/c from each group received 50 L3 of* B. malayi* each into the peritoneal cavity and were euthanized on day 15 after L3 challenge to assess the recovery of developing L3. The remaining 5 mice from each group were kept unchallenged and euthanized on day 30 post infection (p.i.) for investigating the immune responses generated by the recombinant protein.

Similarly* Mastomys* from all the three groups were challenged with 100 L3 of* B. malayi* subcutaneously (s.c.) one week after the final booster dose. Half of the animals from each group were euthanized on day 30 after L3 challenge and the remaining animals on day 180 after L3 challenge, respectively, to investigate the prophylactic efficacy and cellular proliferation in Bm-iPGM in immunized and control animals.

### 2.11. Bm-iPGM Specific Antibody and Isotype Levels in Sera by ELISA

IgG antibody titre and antibody isotypes were measured by ELISA. For measuring IgG antibody titre, the wells of ELISA plate (Nunc, Denmark) were coated with 1 *μ*g/mL of Bm-iPGM (100 *μ*L/well) in carbonate buffer pH 9.6 at 4°C overnight (O/N), blocked (1% gelatin in PBS containing Tween-20) for 2 hours at 37°C, and washed thrice with PBS-T with each single washing for 5 min. Pooled serum of immunized and control group of animals (BALB/c) was used as primary antibody using serial twofold dilutions starting from 1 : 50 to 1 : 102400 while goat anti-mouse IgG-horse radish peroxidase (HRP) was added (1 : 10000) after washing and plate was incubated at 37°C for another 1 h. Reaction was developed by adding OPD substrate prepared fresh (20 mg of OPD in 25 mL citrate buffer of pH 5.0 and 20 *μ*L of H_2_O_2_) in dark for 10–15 minutes at RT and terminated by adding 2.5 N H_2_SO_4_. Absorbance was read at 492 nm in an ELISA plate reader. For antibody isotyping, pooled sera (BALB/c; 1 : 100) was used as primary antibody while goat-anti-mouse monoclonal antibodies to IgM, IgA, IgG1, IgG2a, IgG2b, and IgG3 (1 : 1000) and rabbit anti goat-IgG-HRP (1 : 5000) (Sigma antibody isotype kit, USA) served as secondary and tertiary antibodies, respectively. Reactions were measured after adding the substrate OPD as mentioned above. Mean of the triplicate OD values was calculated and was used for plotting the graph.

### 2.12. Oxidative Burst in Peritoneal Macrophages

Real-time monitoring of intracellular reactive oxygen species (ROS) in peritoneal exudate cells (PEC) of BALB/c was determined through a fluorometric assay using 2′,7′-dichlorofluorescein diacetate (DCF-DA) as described earlier [22] with minor modifications [20]. Briefly, freshly harvested PEC's (from immunized and control animals) at 1 × 10^6^ cells/tube were probe loaded with the DCF-DA at final concentration of 1 *μ*M for 15 min at 37°C in CO_2_ incubator. ROS levels in individual living cells were determined by sequentially measuring their fluorescence intensity (FI) on FACSCalibur (BD, USA). Data was analyzed by CellQuest Software (BD, USA) and mean ROS values were evaluated for cell populations.

### 2.13. Immunophenotyping of T and B Lymphocyte Population

Splenocytes from PBS/adjuvant control and Bm-iPGM immunized groups of BALB/c were used for immunophenotyping to assess lymphocyte subset population on a flow cytometer (FACSCalibur, BD, USA) using fluorochrome (FITC or PE) conjugated anti-mouse antibodies (Serotec, UK) directed against receptors to CD4, CD8, and CD19 [21]. Splenocytes (1 × 10^6^) were initially blocked with Mouse Seroblock FcR at RT for 10 min, washed, and divided into different tubes for labelling with monoclonals to CD4+ and CD8+ T cells or CD 19+ B cells for 10 min at RT. Cells were washed and finally suspended in sheath fluid for analysis by FACSCalibur using CellQuest analysis software (BD, USA) after gating the forward and side-scatter settings to exclude debris. For each determination, 20,000 cells were analyzed and the results are reported as percentage of each cell population.

### 2.14. Intracellular Th1 and Th2 Cytokine Response in Immunized BALB/c

The measurement of intracellular cytokines in the spleen was done as per manufacturer's (BD, USA) protocol as mentioned earlier [21]. Briefly, splenocytes (4 × 10^6^/mL) were incubated with brefeldin A (10 *μ*g/mL) (Serotec, UK) in dark for 6 h at 37°C and reincubated with mouse Seroblock FcR at RT for another 10 min. Cells were washed and incubated with FITC-rat anti-mouse CD4+ antibody. Leucoperm A and Leucoperm B (Serotec, UK) were added at RT for 15 min and cells were dispensed in four tubes each containing 1 × 10^6^ cells/100 *μ*L. PE-rat anti-mouse monoclonal antibodies to cytokines interleukin- (IL-) 2, IL-4, IL-10, and IFN-*γ* were added to separate tubes and cells were finally suspended in 500 *μ*L of 0.5% paraformaldehyde for flow cytometer readings.

### 2.15. Cellular Immune Response in Immunized and Control* Mastomys* Groups

The proliferation of splenocytes isolated from the control and experimental* Mastomys* after vaccination and challenge was performed from both the batches as described earlier [23]. In brief, spleen was aseptically removed and cells were passed through a sterile nylon cell strainer (40 *μ*m pore size; BD Falcon, USA) to prepare single cell suspension. Cells (100 *μ*L/well) from the stock (5 × 10^6^ cells/mL) were plated in a 96-well culture plate in triplicate and stimulated with 100 *μ*L Bm-iPGM (optimal concentration 2.5 *μ*g/mL) or concanavalin A (2.5 *μ*g/mL; Sigma, USA) for 72 h and pulsed with 1.0 *μ*Ci/well of [3H] thymidine (3H-Tdr, specific activity 18 Ci/m mole, BARC, India) for 18 h preceding harvest. The radioactive incorporation in cells was measured in a *β*-counter (Beckman Instruments, Palo Alto, CA) using scintillation fluid. The stimulation index (SI) was assessed as a ratio of mean cpm (counts per minute) values of stimulated and unstimulated cultures.

### 2.16. Effect of Bm-iPGM on Parasitaemia and Parasite Burden in BALB/c and* Mastomys*

The BALB/c mice were euthanized on day 15 after L3 challenge to observe effect of vaccination on development of L3 to L4. On the other hand, half of the* Mastomys* from all the three groups were euthanized on day 30 to assess effect of immunization on development of young adults and remaining half on day 180 after L3 challenge to monitor microfilaraemia as well as effect on the adult worm establishment [12]. Various tissues, namely, heart, lungs, testes, and lymph nodes, were isolated and teased gently in PBS to recover adult worms. Female worms were teased on glass slide in a drop of PBS and observed microscopically to observe the effect of protein on worm fecundity. Data were compared with that of controls and arithmetic means were calculated for blood Mf density, worm burden, and female worm reproductive potential.

### 2.17. *In Vitro* Antibody-Dependent Cellular Adhesion and Cytotoxicity

Adherence of PECs to the surface of Mf and L3 was observed as described earlier [20]. Mf (100) and L3 (10) were individually cocultured with 1 × 10^6^ PECs isolated from normal* Mastomys* in 96-well plate in presence of serum collected from immunized and normal* Mastomys*. Each well contained 100 *μ*L PECs, 50 *μ*L serum (1 : 32), and 25 *μ*L guinea pig serum as a source of complement. Plates were kept at 37°C in a CO_2_ incubator (Binder, Germany) and cell adherence on the surface of parasite and further cytotoxicity was noted microscopically after 1, 3, 6, 24, and 48 h of incubation. Cytotoxicity was expressed by considering the number of immobile or dead parasites by adherence of effector cells against the total number of parasites recovered within 48 h. The percentage of cytotoxicity was calculated by subtracting the number of dead/immobile parasites from the total parasites taken, dividing the result by total number of parasites and finally multiplying by 100. Furthermore, the presence of Bm-iPGM antigen on the surface of Mf and L3 was also investigated by fluorescence microscopy using polyclonal antibody raised against Bm-iPGM. In brief, 10 L3/50 Mf were incubated with pooled serum (1 : 500 dilution) from Bm-iPGM immunized* Mastomys* (collected on day 30) for 4 h at 37°C in 48-well flat-bottom tissue culture plates. The parasites were washed and reincubated with secondary antibody (goat anti-mouse IgG-FITC, 1 : 10000) for 2 h at RT on a rotor-shaker and parasites were finally transferred to glass slide for fluorescence microscopy (Nikon, Japan).

## 3. Statistical Analysis

Data were analyzed using one-way analysis of variance (ANOVA). Individual comparisons following ANOVA were made using the Newman-Keuls method with the help of statistical software PRISM 3.0. Results of flow cytometry and worm recovery have been presented as mean ± S.E. The criterion for statistical significance between the results of immunized and control groups were as follows: *P* < 0.05 was considered as significant, *P* < 0.01 was considered as highly significant, *P* < 0.001 was considered as very highly significant, and *P* > 0.05 was considered as nonsignificant.

## 4. Results

### 4.1. Bm-iPGM is Composed of Two Identical Domains and Is Highly Antigenic

**Figure 1:**
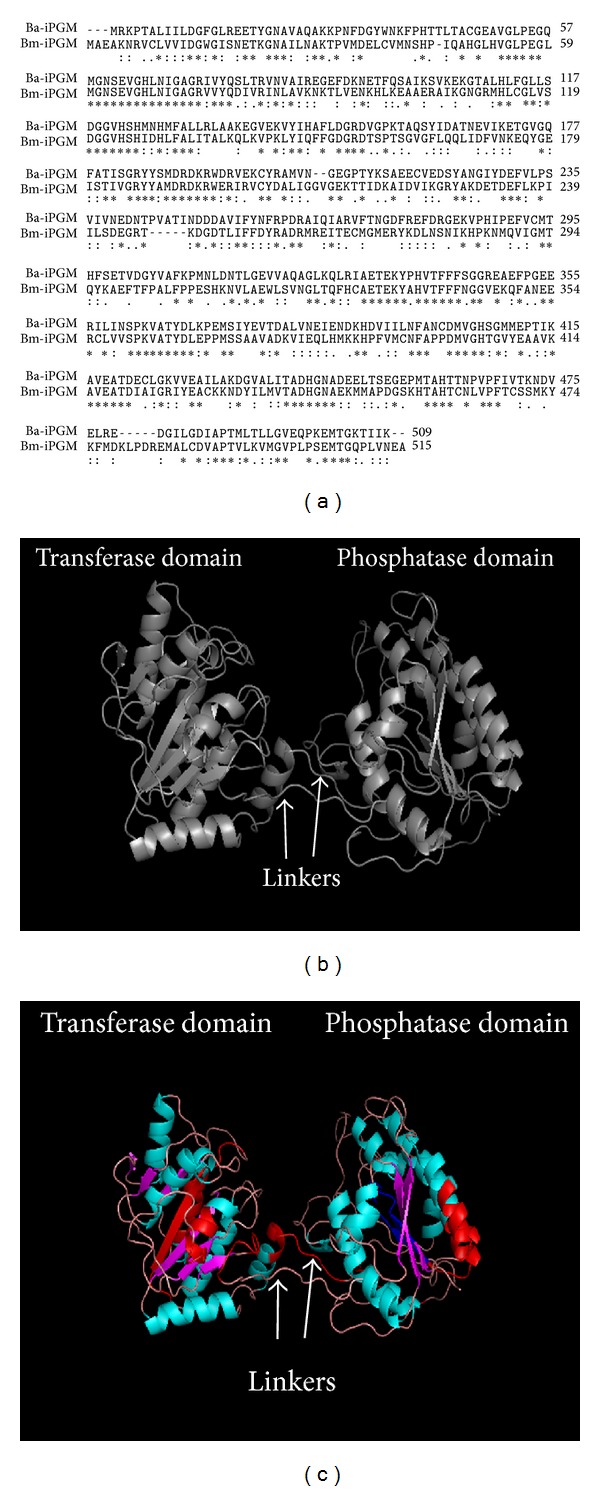
Amino acid sequence alignment and* in silico* structure of Bm-iPGM. (a) Multiple sequence alignment of the deduced amino acid sequence of Bm-iPGM; Bm-iPGM sequence was aligned with* Bacillus anthracis* cofactor-independent 2 phosphoglycerate mutase taken as template (Accession no. 2IFY_A) using Clustal W. Bm-iPGM showed 41% identity with amino acid sequence of* B. anthracis* iPGM. Regions of identity (∗), strong similarity (:), and weak similarity (.) are displayed. (b)* In silico* cartoon structure of Bm-iPGM. Helical content matches with the experimental CD data. (c) Bm-iPGM* in silico* cartoon structure showing quantitatively predicted MHCI and MHCII binding stretches (regions in red are presented by MHCI while the dark blue are presented by MHCII).

Amino acid sequence alignment of Bm-iPGM with iPGM from* B. anthracis* using Clustal W showed 41% identity (Figure 1(a)). Bm-iPGM structure was generated with 100% precision and 41% identity using the same structure of* B. anthracis* cofactor-independent 2 phosphoglycerate mutase taken as template (PDB id: c2ifyA, length: 508 AA). The data analysed by “The PyMOL Molecular Graphics System” showed Bm-iPGM to be composed of two identical domains connected by two linkers. Both the domains show similar folds containing central *β* sheet structure which are flanked on both sides by *α* helices (Figure 1(b)). The* in silico* prediction showed 21 antigenic determinants in the protein with an average propensity being 1.0233 (see Supplementary Table 1 available online at http://dx.doi.org/10.1155/2014/590281). A number of MHC binding peptides were identified for the alleles used in analysis and Table 1 shows the best predicted binding peptide for each allele used in analysis and their log score. These peptides were also visualised and highlighted in the cartoon structure of Bm-iPGM (Figure 1(c)).

### 4.2. Bm-iPGM Was Cloned, Recombinant Protein Optimally Expressed as a Single Band of ~60 kDa

**Figure 2:**
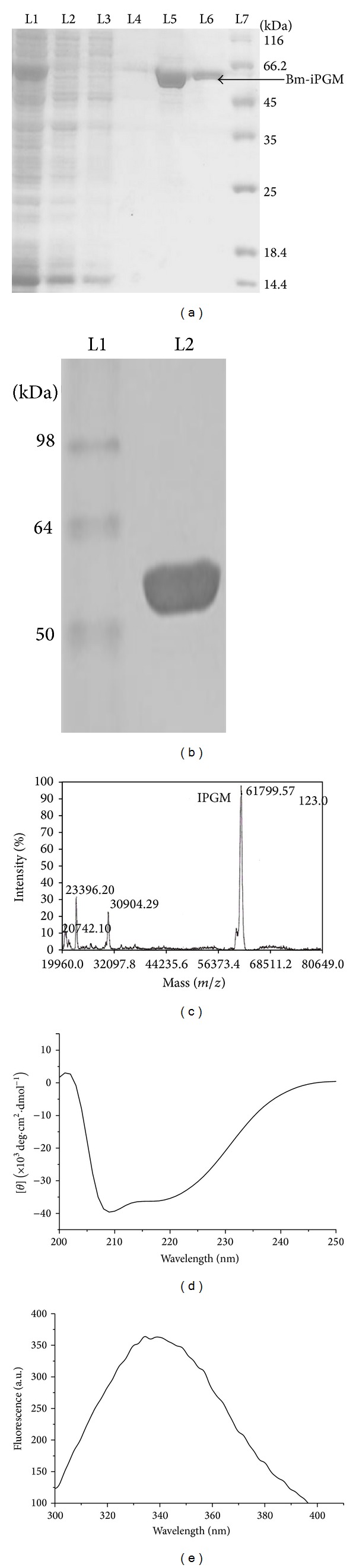
Cloning, expression, and purification of Bm-iPGM. (a) Purification of Bm-iPGM: L1: flow through; L2-L4: wash 1–3; L5 and L6: elute 1-2; L7: standard protein marker (kDa). (b) Western blot analysis using anti-His mAb: L1: prestained protein marker; L2: purified Bm-iPGM. (c) MALDI-TOF analysis of the molecular mass of recombinant Bm-iPGM. A single major peak confirmed the mass of recombinant Bm-iPGM to be 61.799 kDa. (d) Far-UV CD spectra of Bm-iPGM; CD measurements were made on JASCO J810 spectropolarimeter calibrated with ammonium (+)-10-camphorsulfonate with 6 *μ*M protein in 10 mM CGH buffer. (e) Fluorescence emission spectra of Bm-iPGM and spectra of Bm-iPGM in 50 mM phosphate buffer were recorded with Perkin Elmer LS50B luminescence spectrometer. On excitation at 280 nm, maximum emission spectra were noted at 340 nm.

The 1548 bp gene was successfully cloned into pTZ57R/T (2886 bp) vector, gene sequence verified by sequencing, and subcloned into expression vector pET 28a. The maximal protein expression was obtained after four hours of 0.5 mM IPTG induction at 37°C. A ~60 kDa recombinant protein band authenticated the expressed protein to be recombinant protein in-frame with the N-terminal 6x-His Tag (Figures 2(a) and 2(b)) which was found to be biochemically active. The exact mass of recombinant protein was found to be 61.779 kDa as analyzed by MALDI-TOF (Figure 2(c)).

### 4.3. Secondary Structure Analysis by Spectroscopy Revealed Bm-iPGM to Be *α*/*β* Type Protein

Far-UV CD spectrum can be used empirically as “blueprint” of a particular protein, providing information about the polypeptide backbone and the protein conformation in terms of its secondary structure [24]. The secondary structure of Bm-iPGM as characterized by far-UV CD shows that it is *α*/*β* type protein. As depicted in Figure 2(d), Bm-iPGM has two negative peaks around 222 nm and 208 nm and a stronger positive peak near 190 nm, which is a characteristic of predominant *α*-helical protein secondary structure [25]. Analysis of the averaged far-UV CD spectrum gave an estimate of 56.26% *α*-helix and 5.69% *β* strands. The intrinsic fluorescence of Bm-iPGM was studied to disclose the microenvironment surrounding the residues of tyrosine and tryptophan. When excited at 280 nm, the maximum emission of Bm-iPGM was recorded at 340 nm (Figure 2(e)) revealing that tyr and trp residues were mainly located in hydrophobic environment and the Bm-iPGM was purified in its native form.

### 4.4. Bm-iPGM Is Expressed by All the Major Life-Forms of* B. malayi* and Is Excreted Out by Adult Worms

**Figure 3:**
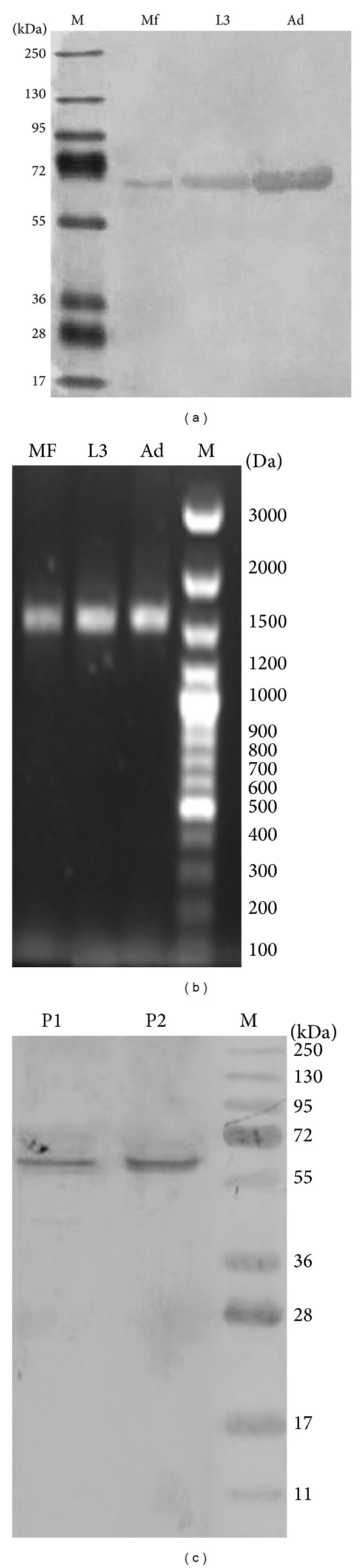
Stage specific expression of Bm-iPGM. (a) Western blot displaying expression of Bm-iPGM. M: standard protein marker; Mf: microfilariae, L3: infective larvae, and Ad: adult parasites. (b) 1.0% agarose gel displaying Bm-iPGM amplification; Bm-iPGM gene was amplified from cDNA of three major life stages of* B. malayi* using specific primers. Mf: microfilariae, L3: infective larvae, Ad: adult parasites, and M: standard DNA marker. (c) Bm-iPGM in excretory-secretory products of adult parasite. Western blot was done to confirm presence/absence of iPGM enzyme in the ES product of* B. malayi*. Anti-Bm-iPGM antibody raised in mouse showed reactivity with the purified recombinant protein as well as the ES product of adult parasite. P1: purified recombinant Bm-iPGM, P2: adult worm ES product.

The polyclonal antibodies raised against the recombinant Bm-iPGM reacted with the native protein in lysates of adult parasites, Mf, and L3 (Figure 3(a)). Bm-iPGM gene was also amplified from cDNA of three major life stages of* B. malayi* using gene specific primers (Figure 3(b)). The results demonstrate presence of Bm-iPGM in all the three life stages analysed, thus demonstrating it to be an abundant protein. Polyclonal antibodies raised against Bm-iPGM reacted with recombinant Bm-iPGM and with the ES product demonstrating it to be present in the* in vitro* excretory-secretory products of female* B. malayi* worms (Figure 3(c)).

### 4.5. Human* W. bancrofti* Patients Harbour Serum IgG Antibodies to Bm-iPGM

**Figure 4:**
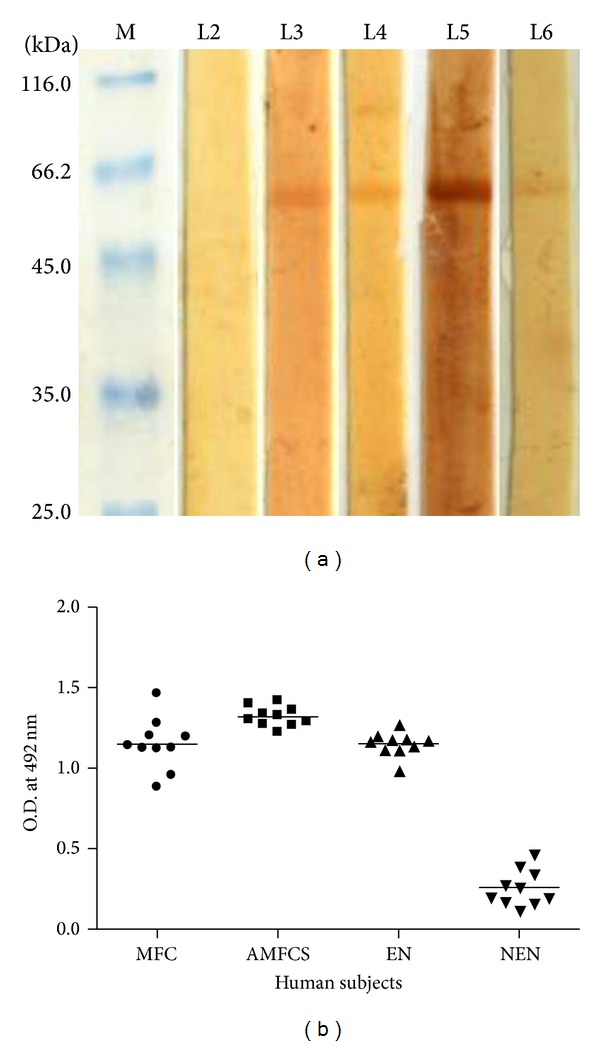
Seroreactivity of Bm-iPGM with human bancroftian sera pooled from 10 filarial patients of each category. (a) Western blots showing cross-reactivity of recombinant Bm-iPGM with various categories of human bancroftian sera. L1: standard protein marker; L2: nonendemic normal; L3: endemic normal; L4: microfilaraemic asymptomatic; L5: microfilaraemic symptomatic; L6 amicrofilaraemic symptomatic patients. (b) Bm-iPGM specific ELISA showing reactivity of Bm-iPGM with human bancroftian sera taken from human subjects belonging to various categories; MFC: microfilaraemic carrier, AMFCS: amicrofilaraemic symptomatic, EN: endemic normal, and NEN: nonendemic normal individuals. Serum (1 : 200) from ten individuals per clinical category was tested in ELISA for reactivity with recombinant Bm-iPGM. All the three groups from filarial endemic area revealed elevated levels of anti-Bm-iPGM antibodies.

The recombinant protein showed good immunoreactivity in Western blot with bancroftian human sera belonging to different clinical categories, namely, nonendemic normals (NEN), endemic normals (EN), asymptomatic microfilaraemic (Mf+ve) carrier, microfilaraemic symptomatic (MFC), and amicrofilaraemic symptomatic (AMFCS), demonstrating the presence of Bm-iPGMin the target human parasite* W. bancrofti *(Figure 4(a)). The individuals from NEN category who are not exposed to filarial larvae did not display any reactivity with the recombinant enzyme showing filarial specificity of the expressed protein.

Bm-iPGM specific IgG ELISA was also carried out to determine the seroreactivity of individual category of serum samples from microfilaraemic, amicrofilaraemic symptomatic, EN, and NEN individuals. All the former three groups analysed revealed elevated levels of anti-Bm-iPGM IgG antibody with amicrofilaraemic symptomatic patients displaying highest antibody titre which was significant over the other groups (*P* < 0.01) (Figure 4(b)). However nonendemic normals did not react to Bm-iPGM.

### 4.6. Bm-iPGM Generates Vigorous Antibody Response in BALB/c Mice

**Figure 5:**
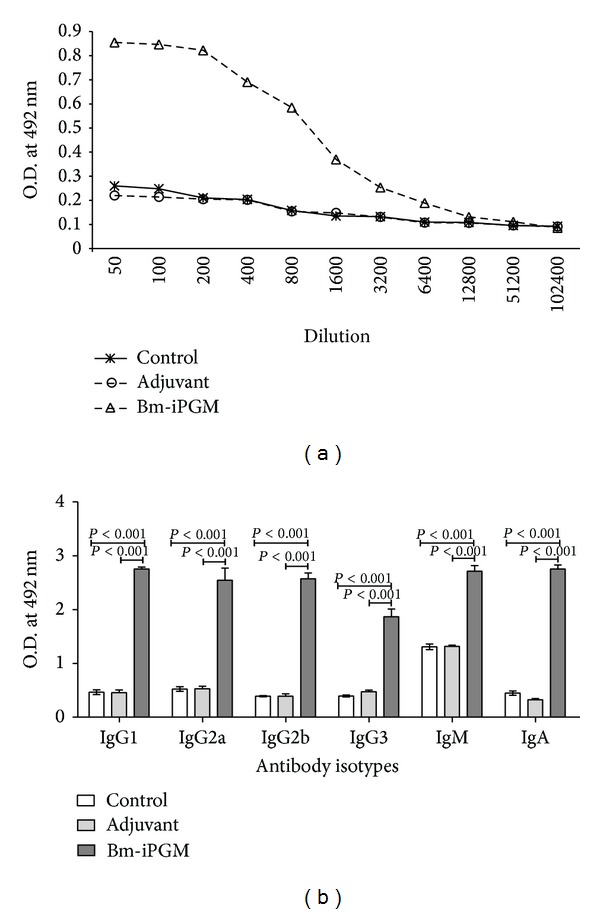
Bm-iPGM specific IgG antibody and antibody isotypes. (a) Antibodies were detected by ELISA in the pooled sera of BALB/c mice administered with Bm-iPGM along with adjuvant and PBS only. Elevated IgG level was maintained in the Bm-iPGM immunized group. (b) Anti-Bm-iPGM antibody isotype levels (IgG1, IgG2a, IgG2b, IgG3, IgM, and IgA) in the pooled sera of Bm-iPGM immunized, adjuvant immunized, and control groups. Considerable increase in the levels of all the isotypes was noticed. Each bar represents mean of triplicate OD values taken at 492 nm each obtained with pooled sera of five experimental animals.

Antibody levels were measured in the sera obtained when the animals were euthanized on day 30 post infection (p.i.). The Bm-iPGM group developed higher levels of Bm-iPGM specific antibodies compared to PBS control and FCA/FIA group. Anti-Bm-iPGM antibody level remained higher in Bm-iPGM group even at 1 : 6400 dilution. None of the controls developed Bm-iPGM specific antibody response (Figure 5(a)). Measurement of Bm-iPGM specific IgG isotypes in experimental groups revealed that animals immunized with recombinant Bm-iPGM induced predominantly elevated level of IgG1, IgG2a, IgG2b, IgG3, IgM, and IgA (Figure 5(b)). IgG2a/IgG1 ratio was indicative of a mixed type of Th1/Th2 immune response. Animals from control groups did not develop Bm-iPGM specific antibody isotypes.

### 4.7. Bm-iPGM Activates the Antigen Presenting Cells (APCs) Upregulating the Production of Reactive Oxygen Species

**Figure 6:**
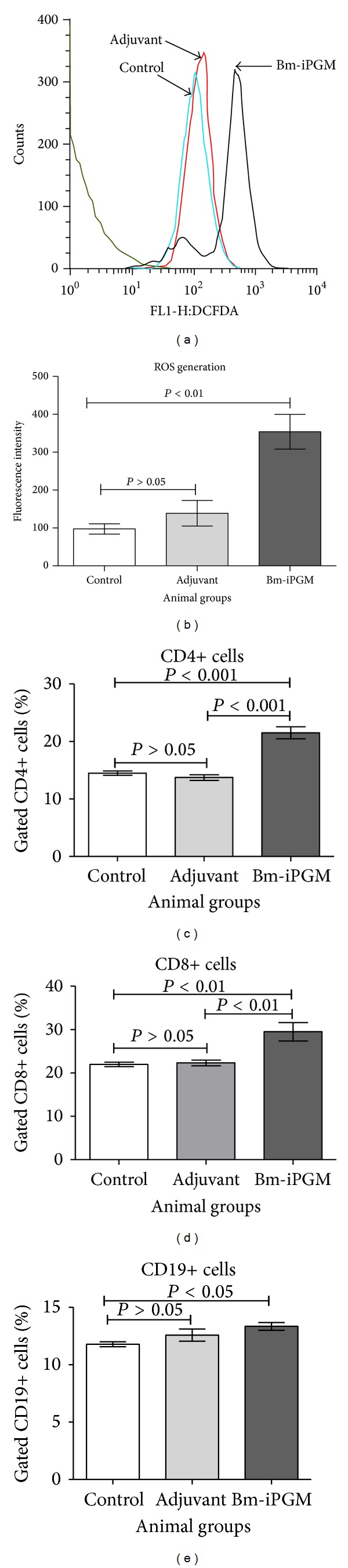
Flow cytometric analysis of ROS generation by peritoneal macrophages and splenic lymphocyte surface staining for T and B cells. (a) Representative FACS histogram for ROS generation from each group is displayed and values closer to mean are represented. (b) Reactive oxygen species produced by the peritoneal macrophages (1 × 10^6^/mL) isolated from for all groups were loaded with probe DCF-DA and ROS generation was evaluated on day 30 when the animals were euthanized. Bm-iPGM immunization led to activation of macrophages which significantly generated reactive oxygen species (*P* < 0.01). (c) CD4+ T cell marker, (d) CD8+ T cell marker, and (e) CD19+ B cell marker. Significant increase was noticed in the number of CD4+ T cells (*P* < 0.001) and CD8+ T cells (*P* < 0.01) from Bm-iPGM immunized animals. Though a marginal expansion in B cell population was observed, it was statistically significant (*P* < 0.05).

A real time monitoring of oxidative burst generated from peritoneal macrophages of immunized and controls group of BALB/c was done. Flow cytometry data indicate that Bm-iPGM immunization led to the generation of significantly higher oxidative burst (*P* < 0.01) in macrophages from Bm-iPGM group as compared to the controls which might have played important role in parasite death (Figures 6(a) and 6(b)).

### 4.8. Increased Number of Both T And B Cell Population Was Observed

**Figure 7:**
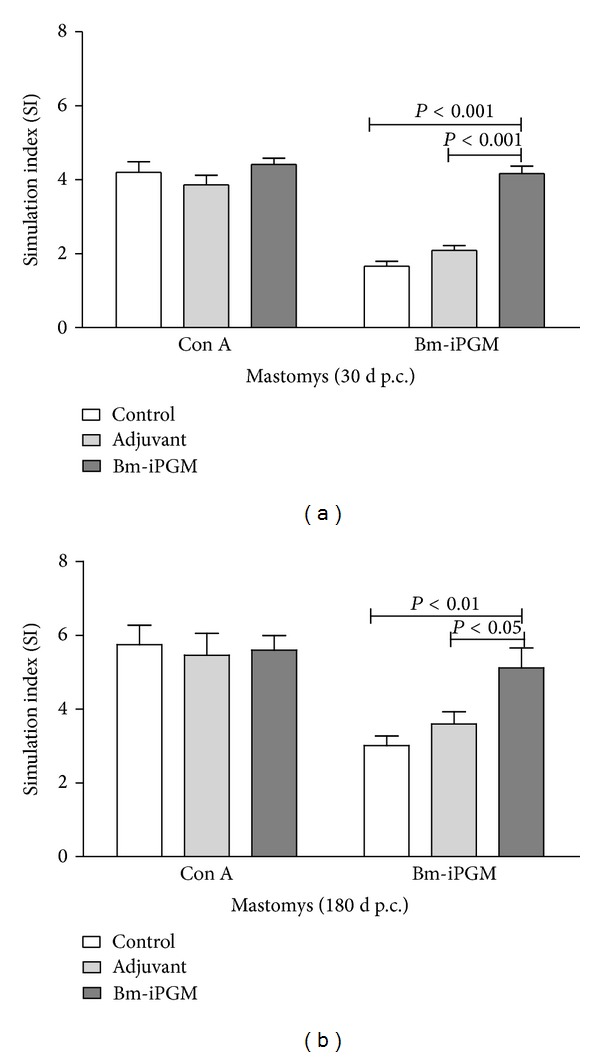
*In vitro* lymphocyte proliferation. Proliferation was assessed in the splenocytes isolated from immunized, adjuvant, and control group of* Mastomys* by radioactive incorporation of [3H]-thymidine after stimulation with either concanavalin A (2.5 *μ*g/mL) or Bm-iPGM (2.5 *μ*g/mL). Radioactive incorporation in the cells was measured and results are expressed as stimulation index. (a) Batch A: euthanized on day 30 after larval challenge. (b) Batch B: euthanized on day 180 after larval challenge.

Bm-iPGM administration into BALB/c mice led to expansion of both cellular and humoral immune response and a significant rise in CD4+ (*P* < 0.001) and CD8+ (*P* < 0.01) T cells (Figures 6(c) and 6(d)). CD 19+ B cell population also increased significantly (*P* < 0.05) (Figure 6(e)). The PBS and FCA/FIA control groups of animals did not showed such heightened cellular and humoral immune response. Experiments were carried out to illustrate the* in vitro* proliferation of splenocytes from both the batches of* Mastomys* euthanized on day 30 and day 180 after larval challenge in presence of Bm-iPGM or mitogen Con A. Spleen cells from Bm-iPGM immunized animals exhibited noticeable proliferation whether stimulated with Con A or Bm-iPGM at both time points (Figures 7(a) and 7(b)).

### 4.9. Immunization with Bm-iPGM Elicits a Mixed Th1/Th2 Immune Response with Marked Reduction in Larval Development in BALB/c While in* Mastomys* Considerably Reduced Microfilarial Density, Adult Worm Recovery, and Female Worm Fecundity Were Observed

**Figure 8:**
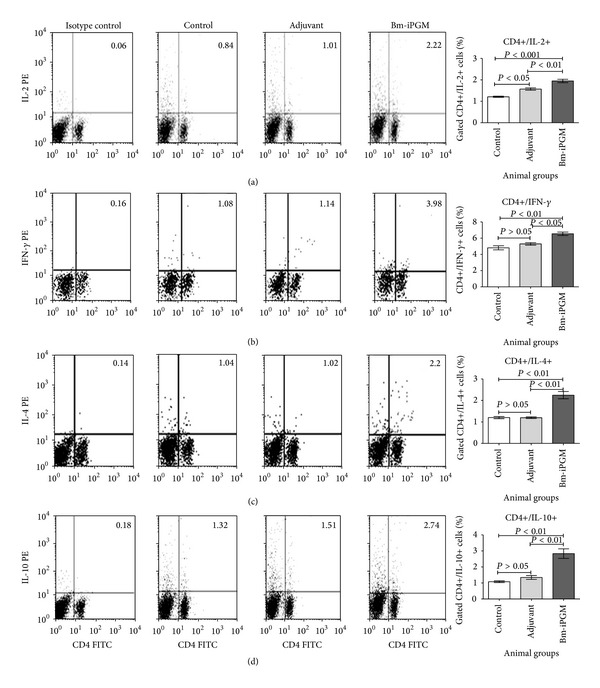
Flow cytometric analysis of intracellular IL-2, IFN-*γ*, IL-10, and IL-4 production in CD4+ T cells. Splenocytes were stained and processed as described in Section 2. Numbers in the upper right quadrant of dot plot represent the mean percentage of CD4+ T cells positive for (a) IL-2, (b) IFN-*γ*, (c) IL-4, and (d) IL-10 in particular group. Bar graph was generated for percentage of CD4+ T cells positive for IL-2, IFN-*γ*, IL-4, and IL-10. Statistical significance of the differences between mean values of immunized and control groups is depicted as *P < 0.05; **P < 0.01; and ***P < 0.001.

**Figure 9:**
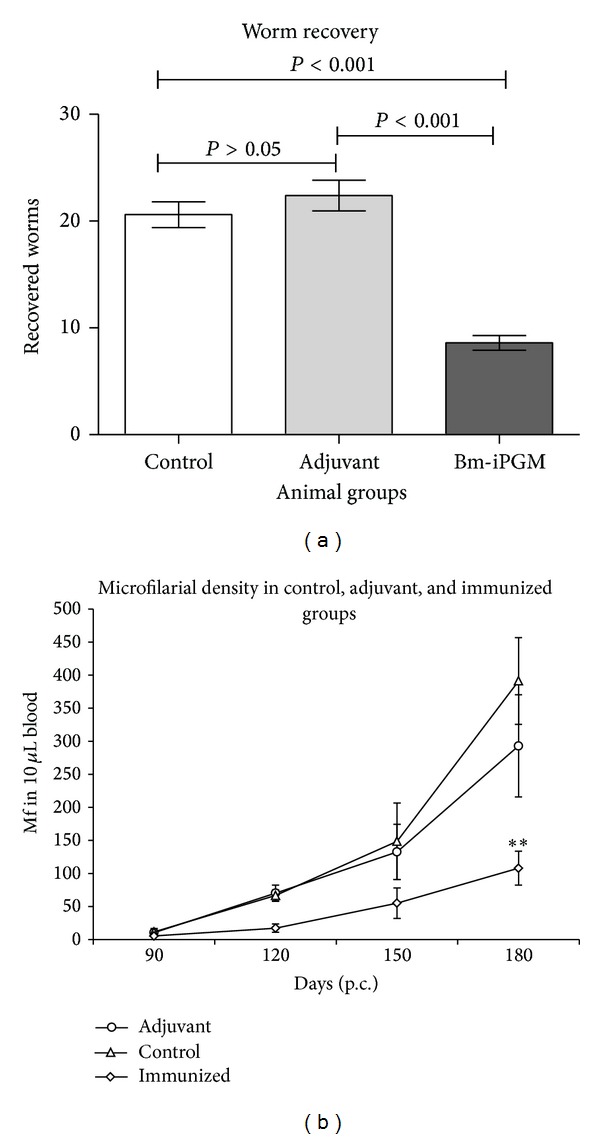
(a) Recovery of* B. malayi* preadults from different groups of Bm-iPGM immunized and control mice. Parasites were collected by washing the peritoneal cavity of infected BALB/c mice. Immunization of mice with Bm-iPGM resulted in marked reduction in worm establishment (*P* < 0.001). Statistical analysis was done using one-way ANOVA followed by Newman-Keuls Multiple Comparison Test. Each bar represents mean ± S.E. of worms recovered from five animals. (b) Assessment of microfilarial density in tail blood of Bm-iPGM immunized adjuvant and control groups. Mf count was initiated from day 90 till day 180 p.c. Control and adjuvant groups exhibited elevated levels of blood Mf density. However, Mf levels remained low in Bm-iPGM immunized group and significant reduction in microfilarial density (*P* < 0.01/72.396%) on day 180 (p.c.) was observed in immunized animals. Each point represents a value obtained from six animals.

The levels of both proinflammatory and anti-inflammatory cytokines were determined intracellularly in the splenic cell population of immunized BALB/c mice. There was an up regulation in the levels of proinflammatory cytokines IL-2 (*P* < 0.001) and IFN-*γ* (*P* < 0.01) as well as anti-inflammatory cytokines IL-4 (*P* < 0.01) and IL-10 (*P* < 0.01) (Figures 8(a), 8(b), 8(c), and 8(d)) as analysed by flow cytometry which indicated generation of a mixed Th1/Th2 immune response. Immunization of BALB/c mice with Bm-iPGM resulted in a significant reduction in worm establishment in Bm-iPGM (number of parasites 8.6 ± 1.1) immunized animals (*P* < 0.001) as compared to the PBS control (number of parasites 20.60 ± 1.2) and FCA/FIA (22.40 ± 1.43) groups. Thus, immunization with Bm-iPGM resulted in up to 58.25% reduction in parasite establishment when BALB/c mice were euthanized on day 15 p.c. (Figure 9(a)).

Immunization of* Mastomys* with Bm-iPGM had profound adverse effect on the Mf density and adult worm establishment when compared with the nonimmunized controls. Though Mf appeared in all the three groups by day 90, (Figure 9(b)) their density was much lower (108 ± 25.34) in Bm-iPGM immunized group in contrast to 391.3 ± 77.32 and 293 ± 100.6 in PBS and adjuvant groups, respectively, at the time of euthanization demonstrating 72.4% reduction over that of control (*P* < 0.05). Considerable reduction (65.45–67.29%) in adult worm recovery was noticed in Bm-iPGM immunized groups when the animals were euthanized on days 30 and 180 after larval challenge (Table 1). In contrast to immunized groups (8–10 worms/animal), the average recovery of adult worms ranged between 24.50 and 28.0 in the two control groups. Vaccination also led to significant adverse effect on the female worm fecundity as observed on day 180 (Table 1). The percentage of sterile adult females recovered from Bm-iPGM gp was 69.97 ± 4.234 which was significantly higher (*P* < 0.001) than that of the normal control gps (PBS 18.50 ± 2.023% and adjuvant gp 20.51 ± 1.543%).

### 4.10. Bm-iPGM Specific Cellular Adherence and Cytotoxicity to Mf and L3

**Figure 10:**
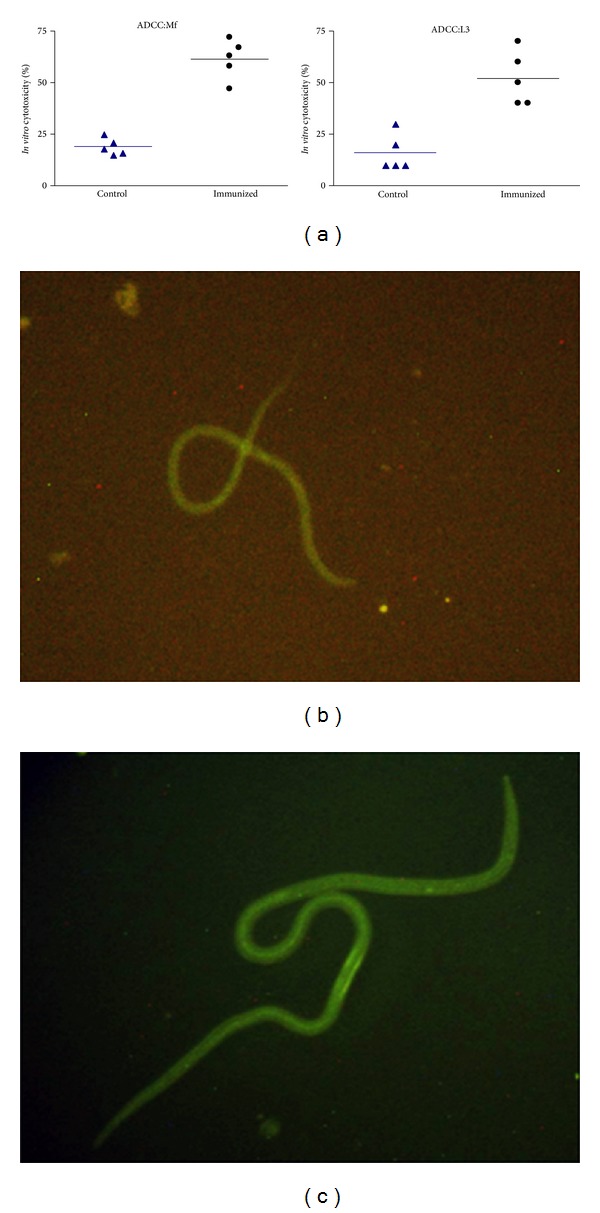
Antibody dependent cellular adhesion to Mf and L3 of* B. malayi.* Ten L3 and 100 Mf were taken per well and were incubated with PEC isolated from normal* Mastomys* in the presence of sera from Bm-iPGM immunized animals. (a) Sera of Bm-iPGM immunized mice promoted adherence of PEC to Mf and L3 larvae and induced significant death of Mf (61.40% cytotoxicity) and L3s (52%). Photographs were captured on phase contrast microscope (Nikon, Japan) at 40x magnification. Data are presented as mean ± S.E. values from five different wells. Interaction of anti-Bm-iPGM antibodies with* B. malayi* Mf (b) and L3 (c) as shown by fluorescence microscopy. Parasites were incubated with anti-Bm-iPGM sera for 4 h and further incubated with FITC labelled anti-mouse IgG for 2 h. Images were captured under fluorescent microscope at 20X for Mf and 10X for L3.

Profound* in vitro* complement mediated cellular adherence and cytotoxicity to both Mf and L3 was noticed (Supplementary Figures 1(a) and 1(b)) in the presence of Bm-iPGM specific antibody. Percentage cytotoxicity was calculated by counting the number of immobile or dead parasites by adherence of effector cells against the total number of live parasites recovered which resulted in 61.4% and 52.0% death of Mf and L3, respectively, (Figure 10(a)) which was statistically highly significant (*P* < 0.001) when compared to normal* Mastomys* serum where no cell adhesion was seen. Interaction of anti-Bm-iPGM antibodies with* B. malayi* Mf (Figure 10(b)) and L3 (Figure 10(c)) was confirmed by fluorescence microscopy.

## 5. Discussion

Phosphoglycerate mutases are the enzyme that catalyzes the reversible interconversion of 3-phosphoglycerate and 2-phosphoglycerate in both glycolysis and gluconeogenesis (Ulrike and Peter, 2007).* B. malayi* possesses cofactor-independent form of PGM while the dependent form is present in mammals that present iPGM as an attractive antifilarial drug or vaccine candidate. Raverdy [17] carried out the biochemical characterization of* B. malayi* iPGM and emphasized its worth as an antifilarial drug target. No information is, however, available on whether this protein plays any role in parasite immunobiology or host-parasite interactions.

In the present investigation, molecular and immune characterization studies of* B. malayi* iPGM have been carried out. Bm-iPGM was cloned, expressed, and purified to homogeneity as a single band protein of ~60 kDa. Recombinant protein was found to be biochemically active in its native form as observed by circular dichroism and fluorescence spectroscopy which demonstrated *α*/*β* type topology having more than 50% *α*-helix and around 5-6% *β* strands which was consistent with the previous report on the three-dimensional crystal structure of iPGM of* B. anthracis* [26]. The homology model predicted the recombinant enzyme to be composed of a globular structure with two domains termed as the transferase and phosphatase interconnected by two short linker peptides. Both the domains demonstrate similar folds containing central *β* sheet structure which are flanked on both sides by *α*-helices, thus further confirming *α*-*β* type topology. Till date, there are no known inhibitors of iPGM and the homology model generated can thus be utilised to design a series of inhibitors providing us with possible antifilarial drugs.

A small fragment of antigen can induce immune response against the whole antigen, thus locating promiscuous binding regions from the whole protein sequence can be useful in designing vaccine candidates. Bm-iPGM gene sequence* in silico* showed presence of 21 antigenic determinants carrying an average antigenic propensity of 1.0284 which points towards the high antigenicity of this protein. Further computational analysis of the target gene sequence predicted binding properties of the peptides to be driven by both MHC I and MHC II immune pathways. The alleles with high frequency within human population and with significant binding data were selected and the best binding peptides for each allele were identified and highlighted on the cartoon structure of Bm-iPGM and these were mainly located in the *α*-helix region of the structure.

Bm-iPGM seems indispensable for the parasite as it was found to be expressed by Mf, L3, and adult parasites and is also present in the excretory-secretory (ES) product of adult parasites. ES products released by live parasites can interfere with every aspect of host immunity [27] and requires functional characterization of their role in parasite and host-parasite interactions. Many of these proteins could serve as drug targets and can also be evaluated for prophylactic efficacy [11]; Bm-iPGM is one such protein and could serve to be major protein targeting all the important parasitic stages.

The serum collected from human bancroftian subjects and endemic normal individuals contained anti-Bm-iPGM antibodies which was demonstrated by reactivity against recombinant Bm-iPGM in blots. In ELISA, amicrofilaraemic symptomatic sera showed higher antibody titre to Bm-iPGM than endemic normals or microfilaraemic carriers while none of the 10 individual sera collected from filarial nonendemic area reacted with Bm-iPGM demonstrating filarial specificity of the recombinant protein which may also find its use in LF diagnosis. Seroreactivity with EN sera is of prime importance because EN are considered to be putatively immunoprotective and despite being continuously exposed to filarial larvae remain infection-free and do not develop the disease [28]. Few such antigens reacting strongly with EN sera have earlier been shown to offer protective immunity [29–32]. We further investigated the nature of immune response generated after administration of recombinant Bm-iPGM in BALB/c mice followed by prophylactic evaluation both in BALB/c and* Mastomys*. It is worth mentioning that BALB/c is immunologically a well dissected model and therefore has been used in the current investigation to decipher the type of immune responses triggered by the recombinant protein while* Mastomys* is a susceptible model that supports establishment of adult parasites mimicking the life cycle of parasite in human host. L3 are the most important stages in the life cycle of filariid that initiate an infection and further establish as adult parasites. Immunized mice were therefore challenged with L3 whose further development into L4 stage or adulthood was observed. Infection of BALB/c with* B. malayi* or* B. pahangi* L3 has earlier provided important insights into host-parasite biology in spite of the nonpermissiveness of immunocompetent mice to* Brugia* species [33].

An active filarial infection is characterised by downregulated Th1 immune response in the form of suppressed T cell proliferation, decreased production of proinflammatory cytokines such as IFN-*γ* and IL-2 along with Th2 dominated profile indicated by increased production of the IgE and Th2 cytokines IL-4, IL-5, IL-10, and IL-13 with expansion and greater mobilization of effector cells such as mast cells, eosinophils, and basophils [27, 34–38]. Treg cells are induced by the parasite to evade the human immune system and are considered to be the important regulators of the immune response to filarial nematodes in experimental animals [39, 40]. CD4+ T cells express increased levels of CD25, CTLA-4, and glucocorticoid-induced TNF receptor family-related gene (GITR) with increased Treg functionality in microfilaraemic individuals. Treatment with antibodies to CD25 and GITR reverses this hyporesponsiveness with reduced parasite establishment [41] while depletion of Treg cells has shown to restore T cell as well as B cell proliferation [42].

Immunization with recombinant Bm-iPGM activated both the cellular and humoral arms of immunity. Profound antibody response was observed in BALB/c with high IgG titers in addition to IgG1, IgG2a, IgG2b, IgG3, IgM, and IgA demonstrating induction of both Th1 and Th2 immune response which was supported by the equal ratio of IgG1 and IgG2a. Antifilarial antibodies have been reported to play an important role in protective immunity evidenced by studies where passive transfer of immune sera from resistant to naive animals showed reduced adult worm establishment [43]. B cell-deficient mice that lacked antibody displayed suppressed vaccine-induced protection against murine filariasis [44]. Antibodies directed against the surface of L3 and Mf have also been shown to be protective and an inverse correlation between adult worms and circulating antibodies has been noticed. IgG is believed to be the predominant antibody involved in antibody dependent cellular cytotoxicity (ADCC) mechanism involving adherence of neutrophils, macrophages, and eosinophils to Mf and L3 [45–48]. In our ADCC experiment peritoneal exudates cells got adhered on the surface of both L3 and Mf in presence of immunized sera causing parasite immobility and death. In a study on* L. sigmodontis* model of filariasis, it has been proposed that the cell recruitment depends on many factors like host susceptibility, immune response, and cell adhesion properties [49]. Immunofluorescence staining of L3 and Mf carried out with serum from immunized* Mastomys* also confirmed attachment of anti-Bm-iPGM antibodies to their surfaces confirming presence of iPGM on parasite surface and its interaction with antibodies. The reduction in parasite recovery and sterilization of recovered female worms from Bm-iPGM immunized animals could be due to this observed cytotoxicity to inoculated L3 which was apparent in BALB/c or both of the groups of* Mastomys* whether euthanized on day 30 after larval challenge or on day 180 p.c.

We observed significant levels of IgA which remains undefined in human bancroftian filariasis, in spite of the fact that studies in other helminths have indicated a protective role for parasite specific IgA restricting infection intensity [50–53]. Recently in human bancroftian filariasis, the role of IgA in protective immunity has been demonstrated [54]. IgA levels in mice have directly been found to be associated with raised interferon gamma (IFN-*γ*) production by T cells [10, 54]. The role of IgM, remains undefined in nematodes; however,* in vitro,* it has been shown to play a major role in adherence of host immune cells to filarial L3 and Mf causing cytotoxicity and their death [55]. Thus, elevated level of Bm-iPGM specific IgM might also have adversely affected the survival of challenged* B. malayi* L3 and their further development in* Mastomys*.

Raised reactive oxygen species (ROS) levels might have been one of the mechanisms responsible for providing protection [56, 57] that could be correlated with the IFN-*γ* levels in the immunized mice since macrophage activation largely depends upon IFN-*γ* produced by Th1 (CD8+T cells). Animals from both the batches of Bm-iPGM immunized* Mastomys* revealed higher T cell proliferation in presence of recombinant Bm-iPGM or mitogen Con A conferring that Bm-iPGM was equally effective in causing cellular proliferation as Con A. Mice deficient in T and B cells have been shown to be permissive to filarial infection, thereby stressing the importance of T and B cells in preventing filarial establishment [58–60]. An expansion in B cell population was also noticed after Bm-iPGM administration in the animals as observed by the increased levels of CD19+ B cells. Low microfilaraemia in antigen immunized group which was apparent from day 120 onwards may also be an attribute for T cell proliferation as state of hyporesponsiveness has been directly linked to high number of circulating Mfs [61]. Significant upregulation in CD4+ and CD8+ cells population was noticed in Bm-iPGM immunized animals, which are in general used for defining helper and cytotoxic T cell subpopulations, respectively, [62, 63]. It is generally believed that a vaccine will have at its core instigation of an antigen specific CD4+ T cell response which plays an important role in development of protective immunity against infection. A number of studies have shown that CD4+ T cells play a critical role in regulating the immune response to nematode parasites where depletion of CD4+ T cells in infected mice has been shown to enhance adult worm and microfilarial burden.

Contradictions to the studies that consider immune responses to helminth parasites including filariids to be Th2 type do exist and reports either impairment of both Th1 and Th2 pathways and domination of Th1 response [64, 65]; these differing observations might have been due to different life stages examined. Past studies in murine models advocate the involvement of both Th1 and Th2 arms of immunity in resistance to filarial parasites [66, 67]; thus, downregulation in Th1/Th2 effector function would certainly facilitate the establishment and maintenance of filarial infections. Therefore, any vaccine regimen that would help to overcome downregulation might be useful in impairing the establishment of filarial parasites.

Immunization with Bm-iPGM led to generation of an effective immune mechanism mediated through an upregulated Th1 (IFN-*γ*, IL-2) and Th2 (IL-4, IL-10) cytokine production that could provide considerable protection (58%: BALB/c; 65–68%:* Mastomys*) against challenged larval development possibly by combating the immune downregulation caused by the challenged larvae. Since BALB/c were euthanized on day 15 after L3 challenge, all the L3 stages had converted into advanced L4 stages and none of the recovered larva was L3. However, this period could demonstrate well that there was noticeable killing of the L3 in Bm-iPGM immunized mice as was observed in the* Mastomys* which displayed reduction in parasite establishment both on day 30 or day 180 p.c.

IL-4 and IL-5 have been shown to play critical roles in the host resistance to* L. loa* infection in knock out BALB/c mice (Nicholas 2012); Th1 cytokine IFN-*γ* controls* B. malayi* infection in murine models; IL-5 controls adult worm development in primary infection and IL-4 mediated pathways are necessary for the control of Mf and the development of adult worms [68]. Studies in IL-4 knockout mice have revealed an undeniable role of IL-4 in countering larval establishment in murine modelof* Litomosoides sigmodontis*, diminished Th2-type responses with failure to produce parasite specific IgG1in* Trichuris muris* infection [69–71]. IL-4 dependent effector mechanisms have been shown to be dependent on IL-10 in mice that were knocked out for IL-4/IL-10 displaying antagonistic activity between IL-4 and IL-10 [68]. Recently, levels of IL-10 have been directly linked to parasite survival, overcome resistance, and allow full patency in murine filariasis [72]. Immunity in human infections has been reported to be associated with an elevated level of IL-2 and IFN-*γ* [73, 74]. EN and chronic patients develop stronger immune response, raised IFN-*γ* level as compared to that of patients carrying active filarial infection [75, 76].* B. malayi* Mf, and L3 have been shown to be killed* in vitro* by IFN-*γ* activated macrophages via production of nitric oxide (NO) and ROS [77–79]. A mixed Th1/Th2 response as observed in the current investigation has been ascribed to exert profound immune protective function [80, 81]. The present findings clearly suggests that, on vaccination with Bm-iPGM, a correct milieu with a mixed type of Th1/Th2 immune response accompanied with innate immunity was maintained which was efficient in providing significant degree of protection against establishment of* B. malayi* in the immunized host. The different molecular events that are required to maintain a balanced cytokine levels need careful investigation to further facilitate vaccine development programme.

In summary, the overall immune response generated by Bm-iPGM correlated with the percentage level of protection achieved in terms of low adult worm recovery, reduced microfilaraemia, and embryostatic effect in female worms. The independent phosphoglycerate mutase of filarial parasite* B. malayi* appears to be an immunogenic protein with diagnostic potential which considerably impairs filarial parasite establishment and presents a promising vaccine candidate. Immunization studies with Bm-iPGM using human administrable adjuvants are underway to further improve its efficacy and usefulness.

## Supplementary Material

Supplementary Figure 1: Antibody dependent cellular adhesion to Mf and L3 of *B. malayi*. Mf and L3 were incubated with peritoneal exudates cells and anti Bm-iPGM sera. Significant cellular adhesion on surface of Mf (A) and L3 (B) was observed that resulted in to significant cytotoxicity to Mf and L3 with in 48 h.
Supplementary Table 1: Prediction of immunodominant T cell antigenic sites from the primary sequence of Bm-iPGM was determined by the programme Pro Pred for both Class I alleles (HLA –A1, HLA-A2, HLA -A0201, HLA -A0205, HLA –A1101, HLA –A3101, HLA –A3302, HLA –B2102, HLA –A3501, HLA –A4403, HLA -5101) and Class II alleles (HLA - DRB1_0101, HLA - DRB1_0301, HLA - DRB1_0401, HLA- DRB1_0701, HLA- DRB1_0801). The table shows the peptides with the best predicted binding affinity for each allele with their log scores.

## Figures and Tables

**Table 1:** Adult parasite recovery and female worm fecundity from control and Bm-iPGM immunized *Mastomys*.

Animal groups	Number of animals	Adult parasite counts/animal		Adult worm recovery (mean ± S.E.)		% reduction in worm burden		% female parasite sterilization
		Day 30 p.c.	Day 180 p.c.	Day 30 p.c.	Day 180 p.c.	Day 30 p.c.	Day 180 p.c.	Day 180 p.c.
PBS	6/6	30, 27, 32, 28, 18, 24	♀24, 25, 15, 14, 21, 20 ♂9, 7, 8, 8, 9, 5	26.50 ± 2.02	27.50 ± 1.95	0.0	0.0	18.50

Adjuvant	6/6	18, 22, 26, 27, 23, 31	♀22, 19, 18, 12, 21, 15 ♂13, 10, 11, 8, 11, 10	24.50 ± 1.83	28.33 ± 2.15	7.54	−3.01	20.51

Bm-iPGM	6/6	8, 8, 10, 5, 9, 12	♀6, 9, 6, 7, 4, 5 ♂4, 3, 3, 2, 6, 2	8.66 ± 0.95***	9.5 ± 0.67***	67.29	65.45	69.97

Statistically significant values were obtained in Bm-iPGM immunised groups as compared to the control groups. ***P < 0.001. Values represented are mean ± S.E.
