# Supplementary material for: Cofactor Independent Phosphoglycerate Mutase of Brugia malayi Induces a Mixed Th1/Th2 Type Immune Response and Inhibits Larval Development in the Host
Source: Biomed Res Int. 2014 Jul 1;2014:590281. doi: 10.1155/2014/590281 (PMC4100390; doi:10.1155/2014/590281)
Supplement: Supplementary file 1 — Supplementary Figure 1: Antibody dependent cellular adhesion to Mf and L3 of B. malayi. Mf and L3 were incubated with peritoneal exudates cells and anti Bm-iPGM sera. Significant cellular adhesion on surface of Mf (A) and L3 (B) was observed that resulted in to significant cytotoxicity to Mf and L3 with in 48 h. Supplementary Table 1: Prediction of immunodominant T cell antigenic sites from the primary sequence of Bm-iPGM was determined by the programme Pro Pred for both Class I alleles (HLA –A1, HLA-A2, HLA -A0201, HLA -A0205, HLA –A1101, HLA –A3101, HLA –A3302, HLA –B2102, HLA –A3501, HLA –A4403, HLA -5101) and Class II alleles (HLA - DRB1_0101, HLA - DRB1_0301, HLA - DRB1_0401, HLA- DRB1_0701, HLA- DRB1_0801). The table shows the peptides with the best predicted binding affinity for each allele with their log scores. [file 590281.f1.doc]

##
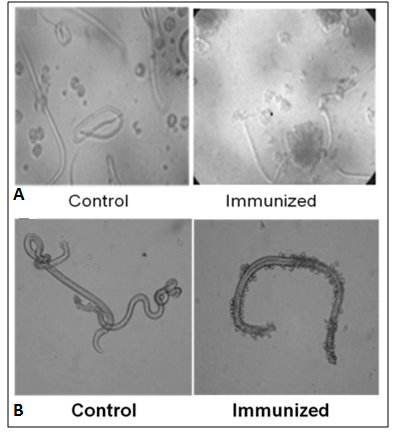


Supplementary Table 1. Predicted antigenic determinants using amino acid sequences of Bm-iPGM

| **No** | **Start Position** | **Sequence** | **End Position** |
| --- | --- | --- | --- |
| 1 | 5 | KNRVCLVVID | 14 |
| 2 | 34 | VMDELCVMN | 42 |
| 3 | 44 | HPIQAHGLHVGL | 55 |
| 4 | 72 | AGRVVYQDIVRINLAVKNK | 90 |
| 5 | 111 | RMHLCGLVSDGGVHSHIDHLFALITALKQLKVPKLYIQ | 148 |
| 7 | 179 | EISTIVGR | 186 |
| 8 | 198 | RIRVCYDALIG | 208 |
| 9 | 218 | KAIDVIK | 224 |
| 10 | 235 | FLKPIIL | 241 |
| 11 | 251 | DTLIFFD | 257 |
| 12 | 300 | FTFPALFPP | 308 |
| 13 | 310 | SHKNVLAEWLSVNGLTQFHCAE | 331 |
| 14 | 334 | KYAHVTF | 340 |
| 15 | 353 | EERCLVVSPKVATYDL | 368 |
| 16 | 372 | MSSAAVADKVIEQ | 384 |
| 17 | 386 | HMKKHPFVMCNF | 397 |
| 18 | 404 | GHTGVYEAAVKAVEATD | 420 |
| 19 | 422 | AIGRIYEA | 429 |
| 20 | 457 | HTAHTCNLVPFTCSS | 471 |
| 21 | 484 | EMALCDVAPTVLKVMGVPL | 502 |

## The antigenicity of Bm-iPGM was determined by Kolaskar and Tongaonkar method. Table shows the 21 predicted antigenic determinants with their sequences and position.
